# Mapping gray matter changes in anorexia nervosa: a functional connectivity network approach

**DOI:** 10.3389/fnut.2025.1667729

**Published:** 2025-09-17

**Authors:** HuCheng Yang, Shu Wang, HuaLiang Li, SiYu Gu, FengMei Zhang, HongHui Wang, ZhenYu Dai, PingLei Pan

**Affiliations:** ^1^Department of Radiology, The Yancheng School of Clinical Medicine of Nanjing Medical University, Yancheng Third People's Hospital, Yancheng, China; ^2^Department of Radiology, Binhai Maternal and Child Health Hospital, Yancheng, China; ^3^Department of Neurology, The Yancheng School of Clinical Medicine of Nanjing Medical University, Yancheng Third People's Hospital, Yancheng, China

**Keywords:** anorexia nervosa, voxel-based morphometry, gray matter, network localization, functional connectivity network mapping

## Abstract

**Background:**

Studies using voxel-based morphometry (VBM) have shown considerable variability in gray matter (GM) changes in anorexia nervosa (AN). However, it remains unclear whether these changes converge on common brain networks underlying the disorder.

**Methods:**

A systematic review was conducted using the PubMed, Embase, and Web of Science databases to identify studies on whole-brain GM alterations in AN published up to October 10, 2024. The Human Connectome Project (HCP) dataset (*n* = 1,093) and functional connectivity network mapping (FCNM) approach to identify common brain networks associated with alterations in AN.

**Results:**

A total of 26 studies involving 667 individuals with AN and 659 healthy controls (HC) were included in this study. Combining the HCP dataset and the FCNM technique, we demonstrated that the disrupted neural networks primarily involved the auditory network, ventral default mode network (DMN), dorsal DMN, and sensorimotor network (SMN). Subgroup analyses further revealed differences in the affected neural networks across specific subgroups, including females-only, adolescents, and adults.

**Conclusion:**

The heterogeneous GM alterations in AN can be attributed to common abnormalities within the auditory network, DMN, and SMN. These disruptions are linked to distorted body image, impaired emotional regulation, and disrupted sensory-motor integration in AN. The FCNM technique provides a unified network-level understanding of the neurobiological mechanisms underlying AN, offering insights for targeted therapeutic strategies.

## Introduction

Anorexia nervosa (AN) is a severe mental illness characterized by aberrant feeding behaviors, an intense desire for thinness, an inability to maintain a minimally normal weight, and obsessive concern with body image (1, 2). Recent global epidemiological reviews indicate that it primarily affects females, with a lifetime prevalence rate of up to 4% among women and approximately 0.3% among men (3). Excessive weight loss in AN can lead to widespread complications, including dysfunctions of the central nervous, cardiovascular, and gastrointestinal systems (4). The etiology of AN is complex, involving a combination of genetic, neurobiological, social-environmental, and psychological factors (1, 5). Clinically, the disorder entails severe medical complications, such as cardiovascular and endocrine dysfunction, and frequent psychiatric comorbidities including depression and anxiety, all of which contribute to its high mortality risk (6–8). This substantial public health burden highlights the need to clarify its underlying neural mechanisms. In the last 20 years, progress in magnetic resonance imaging (MRI) has opened up new opportunities for conducting thorough research on eating disorders (9). An increasing number of neuroimaging studies suggests that AN is associated with significant brain morphological and functional abnormalities (10–13). Despite these advancements, the pathophysiology of AN remains incompletely understood (10, 14, 15).

Voxel-based morphometry, a widely utilized automated technique for the analysis of gray and white matter alterations (16), is considered a key method for investigating the pathophysiological mechanisms of AN. Numerous neuroimaging studies using this technique have identified significant associations between brain GM abnormalities and AN (12, 13, 17, 18). The brain regions with reduced GM in individuals with AN primarily involve the anterior cingulate cortex (ACC), median cingulate cortex, posterior cingulate cortex, inferior frontal gyrus, frontal operculum, superior temporal gyrus, middle temporal gyrus, fusiform gyrus, inferior parietal cortex, occipital cortex, precentral gyrus, precuneus, cerebellum, striatum, and thalamus (11, 12, 18–24). While one recent study reported increased GM in specific brain regions, including the left orbitofrontal gyrus rectus, bilateral fusiform gyrus, bilateral hippocampus, right insula, and bilateral parahippocampal gyrus (25). Conversely, another study observed no significant reductions in GM (26). However, these inconsistent findings limit their utility for elucidating the neurobiological mechanisms of AN. Recent research has highlighted a strong correlation between the onset of AN and disruptions in brain networks, including basal ganglia network, sensorimotor network (SMN), Limbic network, visuospatial network, and default mode network (DMN) (21, 27–30). While coordinate-based meta-analysis (CBMA) has traditionally been used to consolidate diverse findings into common regions (31), accumulating evidence indicates that neuropsychiatric symptoms and diseases may be more precisely mapped to distinct brain networks than to isolated regional abnormalities (32–35). The significance of this network-based approach lies in the fundamental principle that focal structural lesions, such as GM alterations, are not functionally isolated; instead, they compromise the integrity and function of the entire large-scale network in which they are embedded (36, 37). Functional connectivity network mapping (FCNM) is a validated approach that integrates regions of interest with large-scale human connectome data to reveal the intricate relationships between different brain areas, facilitating a deeper understanding of how these connections influence cognitive processes and behaviors (38, 39). This technique can map heterogeneous neuroimaging findings to common neuroanatomical networks and identify disease-specific and symptom-specific brain networks (40). The FCNM method has effectively mapped various neurological and psychiatric symptoms to distinct brain networks (39, 41–46). Despite significant advancements in its application to other diseases, the FCNM approach has yet to be used to explore how focal GM alterations in AN impact brain function from a unified network-level perspective.

**Figure 1:**
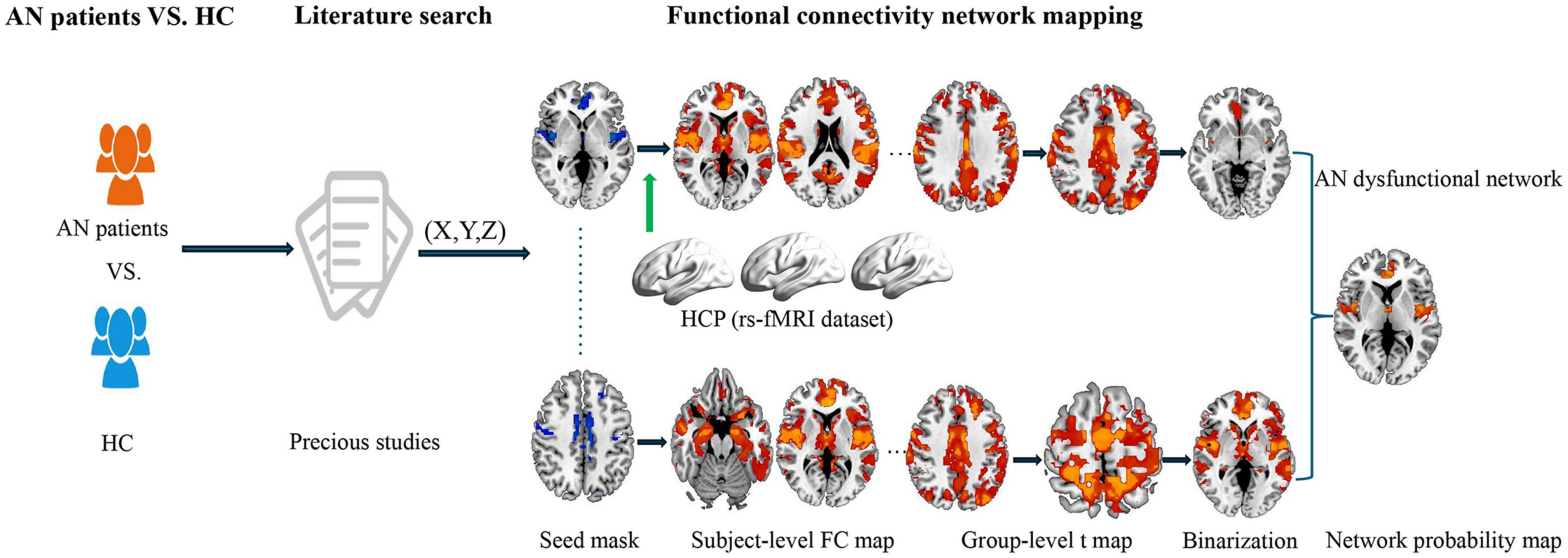
Study design and analysis pipeline. Our primary method entailed integrating published literature to determine the brain regions demonstrating alterations in GM between individuals with AN and HC. By integrating these impacted brain locations with large-scale human connectome data from HCP, we subsequently employed the FCNM approach to identify abnormal brain networks associated with AN. Specifically, spheres centered at each coordinate of a contrast were initially constructed and amalgamated to produce a contrast-specific integrated seed mask. Second, in accordance with the HCP dataset, we calculated a contrast seed-to-whole brain resting-state FC map for each participant. Third, the subject-level resting-state FC maps were subjected to a voxelwise one-sample t-test to determine brain regions functionally associated with each contrast seed. Fourth, the resulting group-level t maps were thresholded and binarized at *p* < 0.05 corrected for multiple comparisons using a voxel-level false discovery rate method. Finally, the binarized maps of GM contrasts were overlaid to produce a network probability map, which were thresholded at 50% to yield AN GM dysfunctional network. AN, anorexia nervosa; HC, health controls; GM, gray matter; HCP, Human Connectome Project; FCNM, functional connectivity network mapping; FC, functional connectivity; rs-fMRI, resting-state functional magnetic resonance imaging.

To address this question, we utilized the FCNM approach to identify brain networks implicated in the structural abnormalities of AN. A schematic representation of the study design and analysis pipeline is illustrated in Figure 1. We hypothesized that the heterogeneous alterations in GM across different brain regions in individuals with AN could be mapped onto common brain networks. The results hold the potential to reconcile the heterogeneous whole-brain GM changes reported in previous VBM studies through a network perspective.

## Methods

### Literature search and selection

Following the PRISMA guidelines (47), we conducted a comprehensive and systematic search of the PubMed, Embase, and Web of Science databases to identify studies on GM changes in AN, published up to October 10, 2024. The search included keywords such as “voxel-based morphometry” OR “VBM” OR “gray matter” OR “gray matter” AND “anorexia nervosa” OR “eating disorders.” Additionally, a manual search of reference lists from relevant reviews and meta-analyses was performed to identify any potentially overlooked studies. The flow diagram in Supplementary Figure S1 details the study selection process.

All studies were included according to the following criteria: (1) published as an original article in a peer-reviewed English-language journal; (2) studies explored GM changes in individuals with AN compared to healthy controls; (3) voxel-wise analyses were conducted at the whole-brain level; (4) studies reported results in Talairach or Montreal Neurological Institute (MNI) space. For longitudinal studies, only baseline data were included in this study.

Exclusion criteria were as follows: (1) lacking a reported coordinate system; (2) only use of region of interest analysis; (3) all coordinates reported were located outside the gray matter mask; (4) absence of relevant comparisons between patients with AN and healthy controls; (5) studies involving animal experiments; (6) reviews or meta-analyses. Only the study with the largest sample size and most comprehensive information was included in the analysis to prevent data duplication from overlapping patient samples in multiple publications. The corresponding author of each study was contacted via email for any necessary additional information. Two investigators, Y. H. C. and W. S., conducted literature searches, selected relevant articles, and independently extracted data. Any discrepancies were discussed with another investigator (P. P. L) until they were resolved. Furthermore, we systematically evaluated the methodological quality of the neuroimaging protocols in all studies incorporated in the current analysis based on a 10-point checklist (48, 49) (Supplementary Table S1).

### Functional MRI data acquisition and preprocessing

Data utilized in this study were extracted from the Human Connectome Project (HCP) 1,200 Subjects Release (S1200), encompassing imaging data of healthy adults aged 22–37 years. A total of 1,093 participants (594 female; mean age = 28.78 ± 3.69 years, SD) constituted the final study sample. Exclusion criteria utilized during HCP data collection encompassed MRI contraindications, existing psychiatric/neurological disorders, recent psychiatric medication intake, pregnancy, and a prior history of head trauma. Demographics of this cohort are detailed in Supplementary Table S2. All coordinates extracted from the previous studies were uniformly converted to the MNI standard space. If an original study reported coordinates in Talairach space, established conversion tools^1^ were used to transform them into MNI space.

High-resolution imaging data for the HCP dataset were acquired on a 3 T Siemens Trio MRI scanner, enabling detailed analyses (specific fMRI parameters are provided in Supplementary Table S3). Participants were excluded on account of poor scan quality, such as significant artifacts or incomplete brain coverage.

Subsequently, the resting-state fMRI data were preprocessed using SPM12^2^ and DPABI^3^. Initial processing involved discarding the first 10 volumes from each participant’s scan to ensure signal stability and participant adaptation to the scanner environment. Subsequently, the remaining volumes underwent slice-timing correction to account for acquisition time differences between slices. Then, realignment was conducted to correct motion across time points, with head motion parameters calculated to determine translation and angular rotation for each volume. All participants met the predefined motion criteria (maximum translation < 2 mm, maximum rotation < 2°). We also computed framewise displacement to quantify inter-volume head motion. Subsequently, to mitigate the effects of physiological noise and motion artifacts, the following nuisance covariates were regressed from each participant’s preprocessed blood oxygenation level dependent time series: linear drift, the 24 motion parameters derived from the Friston model, spike volumes (FD > 0.5 mm), and the mean signals from global tissue, white matter, and cerebrospinal fluid. Global signal regression was included in the resting-state fMRI preprocessing pipeline, as this step has been shown to enhance system-specific correlations and improve functional connectivity estimation. Subsequently, the preprocessed functional data were bandpass filtered (0.01–0.1 Hz). Normalization involved first co-registering individual structural images to their corresponding mean functional image. These co-registered structural images then underwent segmentation and normalization to MNI space. Next, every filtered functional volume underwent spatial normalization to MNI space. The volumes were then resampled to 3-mm isotropic voxels. A Gaussian kernel with a full width at half maximum of 6 × 6 × 6 mm^3^ was applied for spatial smoothing of all data.

### Functional connectivity network mapping (FCNM)

By utilizing the FCNM technique, we aimed to determine whether the diverse GM changes observed in AN are linked to a specific set of large-scale functional brain networks (35, 37, 40, 50–52). To create a contrast seed, 4-mm radius spheres were individually centered at each coordinate of a contrast and then amalgamated. We then performed seed-based functional connectivity (FC) analysis for each participant using the preprocessed HCP resting-state data. The calculation of Pearson correlation coefficients involved comparing the time series of the contrast seed with that of all other brain voxels, and subsequently Fisher-Z transformed to approximate a normal distribution, yielding individual FC maps. Third, the FC maps of 1,093 subjects were examined through a voxel-wise one-sample *t*-test to identify brain regions associated with each seed. Our analysis concentrated exclusively on positive FC, given the ongoing debate surrounding the interpretation of negative connectivity. Following thresholding, the group-level t maps were binarized at *p* < 0.05, with correction for multiple comparisons using a voxel-level false discovery rate method. Finally, binarized connectivity maps derived from each GM contrast were merged to form a network probability map, which was subsequently thresholded at 50% based on previous well-validated FCNM studies (38, 41) to outline the AN GM dysfunctional network.

### Association with canonical brain networks

To enhance interpretability, we examined the spatial relationships between the dysfunctional AN brain networks and 14 established canonical brain networks. These networks include the auditory network (including bilateral superior temporal gyrus and right thalamus), basal ganglia network (including bilateral inferior frontal gyrus, bilateral caudate nucleus and bilateral putamen), language network (including left inferior frontal gyrus/Broca’s area, left middle temporal gyrus, and left supramarginal gyrus), SMN (including bilateral precentral gyrus, bilateral postcentral gyrus and bilateral supplementary motor area), primary visual network (including bilateral cuneus, bilateral lingual gyrus and pericalcarine cortex), dorsal DMN (including bilateral inferior parietal lobule, dorsomedial prefrontal cortex and bilateral posterior cingulate cortex), ventral DMN (including medial temporal lobe, hippocampus/parahippocampal gyrus and ventromedial prefrontal cortex), left executive control network (LECN)[including left dorsolateral prefrontal cortex, left superior parietal lobule and left supramarginal gyrus], right executive control network (RECN) [including right dorsolateral prefrontal cortex, right superior parietal lobule and right supramarginal gyrus], high visual network (including lateral occipital cortex, tempo-occipital junction and fusiform gyrus), visuospatial network (including superior parietal lobule, inferior parietal lobule and superior frontal sulcus), anterior salience network (including bilateral insula, bilateral ACC and bilateral middle frontal gyrus), posterior salience network (including bilateral posterior insula, bilateral posterior cingulate cortex and bilateral supramarginal gyrus), and the precuneus network [including precuneus, part of the parietal cortex and part of the posterior cingulate cortex (53)]. We quantified the spatial relationships by calculating the ratio of overlapping voxels between each dysfunctional AN network and its corresponding canonical network, relative to the total number of voxels within the canonical networks.

### Subgroup analyses

We conducted subgroup analyses on the included samples, dividing participants into female-only, adolescent, and adult subgroups to explore potential differences in brain network abnormalities across these groups.

## Results

### Included studies and sample characteristics

A total of 842 candidate articles were initially identified and subjected to a thorough screening process. Ultimately, 26 studies including data from 667 individuals with AN and 659 healthy controls were included in the analysis (17, 24, 54–66). Subsequently, we conducted a planned subgroup analysis (female-only group, adolescent group, adult group). The female-only group comprised 598 women with AN and 593 healthy controls. The adolescent group included data from 215 individuals with AN and 189 healthy controls. The adult group consisted of 452 individuals with AN and 470 healthy controls. The sample and imaging characteristics of the studies included are outlined in Table 1.

**Table 1:** Sample and imaging characteristics of the studies included AN analysis.

Study	AN/HC	Mean Age SD	Gender female	Age group	AN state	Illness duration months	Scanner	Software	Threshold	Quality score*
Muhlau et al. (2007) (63)	22/37	27.2	100%	Adult	Recovered	116	1.5 T MRI	SPM2	*p* < 0.1 uncorrected	9.5
Castro Fornieles et al. (2009) (60)	12/9	14.5 ± 1.5	91.6%	Adolescent	NA	NA	1.5 T MRI	SPM5 FSL	*p* < 0.5 FWE	10
Suchan et al. (2010) (23)	13/14	26.8 ± 8.4	100%	Adult	Chronic	66.0 ± 60.0	1.5 T MRI	SPM5	*P* < 0.5 FWE	9.5
Boghi et al. (2011) (129)	21/27 10/13 11/14	29.0 ± 10.0 21.4 ± 2.5 35.9 ± 9.25	100% 100% 100%	Adult Adult Adult	Recovered/Acute Acute Chronic	135.6 ± 145.2 22.8 ± 15.6 237.6 ± 133.2	1.0 T MRI NA 1.0 T MRI	SPM2	*P* < 0.5 FDR NA *p* < 0.01 uncorrected	9
Brooks et al. (2011) (66)	14/21	26.0 ± 1.9	100%	Adult	NA	110.4 ± 22.8	1.5 T MRI	SPM5	NA	10
Gaudio et al. (2011) (62)	16/16	15.2 ± 1.7	100%	Adolescent	Acute	5.3 ± 3.2	1.5 T MRI	SPM2	*P* < 0.01 uncorrected	9.5
Joos et al. (2011) (65)	5/18 12	29.6 ± 5.1 25.0 ± 4.8	100% 100%	Adult	Recovered	86.4 ± 72 NA	3.0 T MRI	SPM8	*P* < 0.01 uncorrected	9
Friederich et al. (2012) (64)	12/14 13	24.3 ± 6.2 25.0 ± 4.8	100% 100%	Adult Adult	Acute Recovered	75.6 ± 52.8 68.4 ± 43.2	3.0 T MRI	SPM5	*P* < 0.5 corrected *P* < 0.5 uncorrected	9.5
Mainz et al. (2012) (130)	19/19	15.7 ± 1.5	100%	Adolescent	Recovered	NA	3.0 T MRI	SPM5	FWE corrected	9
Amianto et al. (2013) (59)	17/14	20.0 ± 4.0	100%	Adolescent	Acute	13.0 ± 8.0	1.5 T MRI	FSL	*P* < 0.05 TFCE	9.5
Frank et al. (2013) (55)	19/24	23.1 ± 5.8	100%	Adult	Acute	NA	3.0 T MRI	SPM8	*P* < 0.5 FWE	9
Fonville et al. (2014) (54)	31/31	23.0	NA	Adult	NA	84.0 ± 120.0	1.5 T MRI	FSL	*P* < 0.5 FWE	9.5
Bär et al. (2015) (131)	26/26	23.0 ± 5.0	88.4%	Adult	Acute	22.4 ± 14.8	1.5 T MRI	SPM8	*P* < 0.01 uncorrected	10
Bomba et al. (2015) (61)	11/8	13.6 ± 2.8	100%	Adolescent	NA	14.5 ± 10.9	1.5 T MRI	SPM5	*P* < 0.5 FWE	9.5
D’Agata et al. (2015) (58)	21/17	21.0 ± 5.0	100%	Adolescent	Acute	NA	1.5 T MRI	FSL	*P* < 0.05 uncorrected	9
Fujisawa et al. (2015) (56)	20/14	14.2 ± 1.8	100%	Adolescent	NA	23.6 ± 17.0	3.0 T MRI	SPM8	L_IFG: *P* < 0.05 FWE R_IFG: *p* < 0.05 uncorrected	9.5
Van Opstal et al. (2015) (20)	10/11	22.1 ± 3.3	100%	Adult	NA	42.5 ± 27.6	3.0 T MRI	FSL	NA	9
Björnsdotter et al. (2018) (17)	25/25	20.3 ± 2.2	100%	Adolescent	NA	49.7 ± 42.5	3.0 T MRI	SPM8	*P* < 0.5 corrected	10
Kohmura et al. (2017) (19)	23/29 23	28.5 ± 6.7 NA	100%	Adult Adult	NA NA	126 ± 74.4	3.0 T MRI	SPM8	*P* < 0.5 FWE	9.5
Martin Monzon et al. (2017) (18)	26/20	16.5 ± 0.3	100%	Adolescent	NA	NA	3.0 T MRI	SPM12	*P* < 0.5 FDR	9
Nickel et al. (2017) (132)	34/41	23.8 ± 4.3	100%	Adult	Acute	79.2 ± 44.4	3.0 T MRI	SPM12	*P* < 0.5 FWE	9.5
Phillipou et al. (2018) (24)	26/27	22.8 ± 6.7	100%	Adolescent	Acute	6.4 ± 7.4	3.0 T MRI	SPM12	*P* < 0.5 FWE	10
Oliva et al. (2020) (13)	15/15	25.9 ± 6.2	100%	Adult	Recovered	38.4 ± 38.0	1.5 T MRI	SPM12	NA	9.5
Mishima et al. (2021) (12)	35/35	36.3 ± 10	100%	Adult	NA	15.7 ± 9.0	3.0 T MRI	FSL	*P* < 0.5 FWE	9
Lenhart et al. (2022) (57)	22/18	15.2 ± 1.2	100%	Adolescent	Acute	9.4 ± 6.8	3.0 T MRI	SPM12	*p* < 0.5 FWE	9.5
Tose et al. (2024) (11)	103/102	33.11 ± 12.17	100%	Adult	NA	NA	3.0 T MRI	SPM12	*P* < 0.05 FWE	9.5

AN, anorexia nervosa; HC, health controls; FDR, false discovery rate; FWE, family-wise error; TFCE, Threshold-Free Cluster Enhancement; NA, not applicable; SPM, Statistical Parametric Mapping; FSL, FMRIB Software Library; FWHM, Full Width at Half Maximum. *Ten points in total.

### Convergent aberrant FC associated with GM alterations in AN

**Figure 2:**
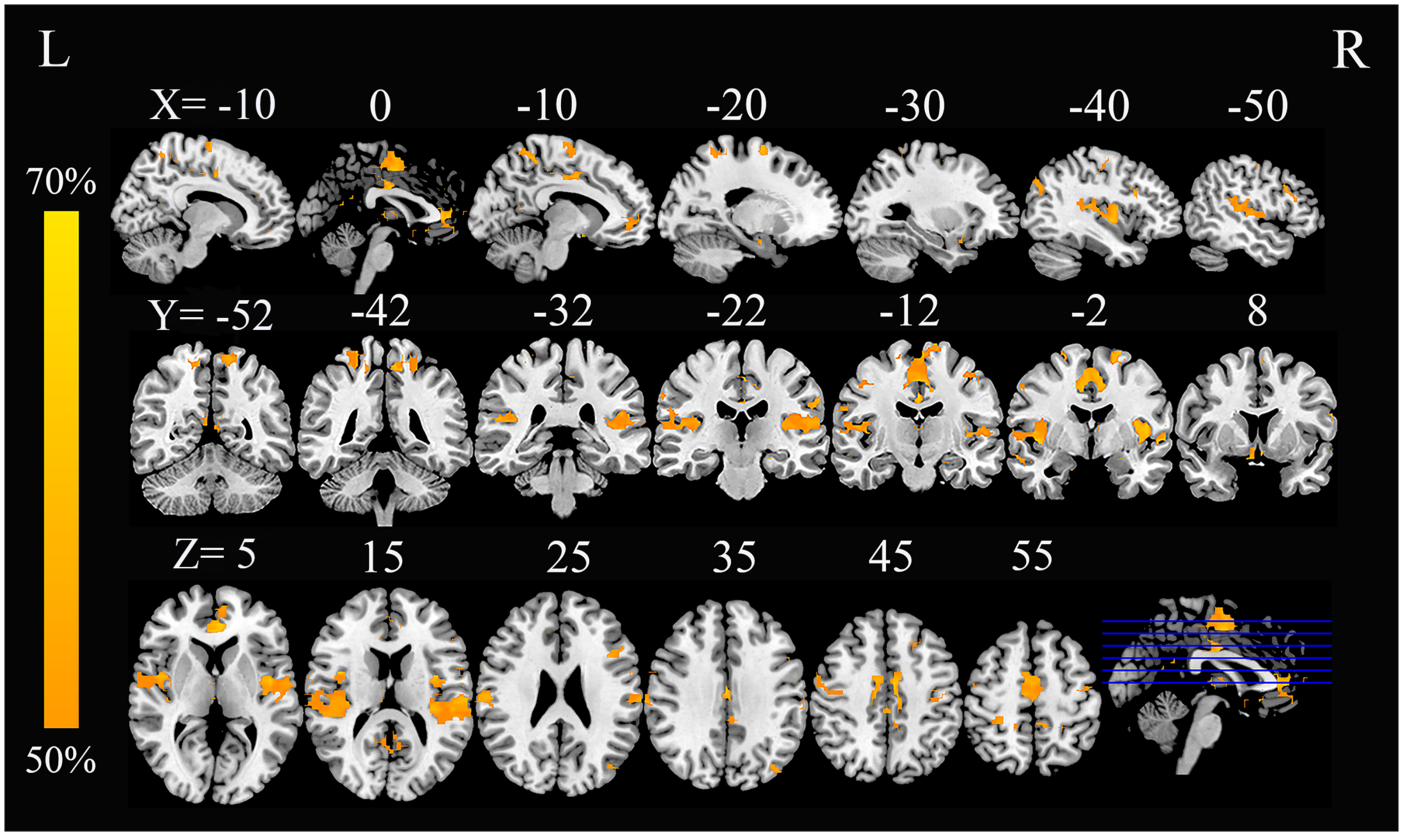
FC overlap maps based on 4-mm radius sphere in AN. Dysfunctional brain networks are shown as FC probability maps thresholded at 50%, showing brain regions functionally connected to more than 50% of the contrast seeds. AN, anorexia nervosa; FC, functional connectivity; L, left; R, right.

**Figure 3:**
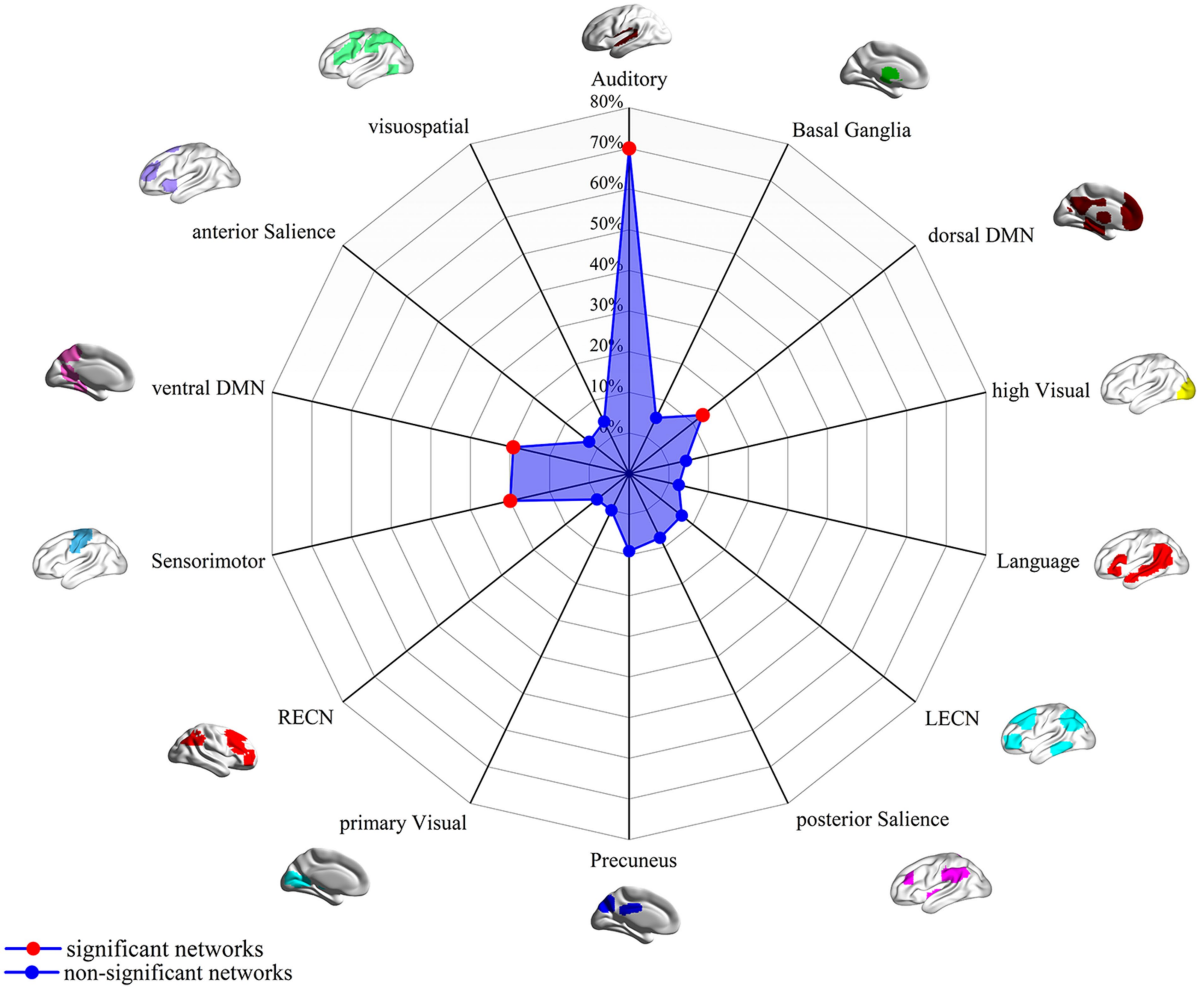
FC overlap maps based on 4-mm radius sphere in association with canonical brain networks. Polar plots illustrate the proportion of overlapping voxels between each AN dysfunctional network and a canonical network to all voxels within the corresponding canonical network. Note: The red dot represents brain dysfunction networks, defined as significant networks, exhibiting ≥ 10% overlap with canonical networks, whereas the blue dot represents non-significant networks with <10% overlap. DMN, default mode network; FC, functional connectivity; LECN, left executive control network; RECN, right executive control network.

In this study, by combining the FCNM approach and large-scale human brain connectome data obtained from the HCP, we identified that the convergent aberrant FC associated with GM alterations in AN involved widely distributed brain regions primarily including the bilateral superior temporal gyrus, right Rolandic operculum, right middle temporal gyrus, bilateral precuneus, bilateral ACC, and bilateral precentral gyrus (Figure 2 and Table 2). FCNM calculations were further conducted using spheres with 1-mm (Supplementary Figure S2) and 7-mm radii (Supplementary Figure S3), respectively, revealed similar brain regions to those obtained with a 4-mm radius sphere. Regarding canonical brain networks, the AN-associated network showed the highest overlap with the following networks: the auditory network includes brain regions such as the bilateral superior temporal gyrus and right Rolandic operculum, with an overlap proportion of 70.5%; the ventral DMN primarily includes the right middle temporal gyrus and bilateral precuneus, showing an overlap proportion of 19.2%. The dorsal DMN mainly involves the bilateral ACC, with an overlap proportion of 13.1%; and the SMN being primarily associated with the bilateral precentral gyrus, with an overlap proportion of 20.6% (Figure 3). The overlap proportions with other canonical brain networks were all below 10%. Then, replicating the FCNM procedure involved spheres with radii of 1-mm (Supplementary Figure S4) and 7-mm (Supplementary Figure S5), the resulting patterns of network overlap closely resembled those generated using the 4-mm radius sphere.

**Table 2:** Brain FC with altered gray matter in AN.

Brain regions	R/L	Voxels	Brodmann areas	*X*	*Y*	*Z*
Precuneus	L	100	BA7	-11	−56	25
Precuneus	R	43	BA7	14	−56	24
Precentral gyrus	L	37	BA6	−32	−12	61
Precentral gyrus	R	115	BA6	40	−12	42
Superior temporal gyrus	L	261	BA22	−46	−8	−15
Superior temporal gyrus	R	148	BA22	44	−4	15
Middle temporal gyrus	R	42	BA21	61	−7	−15
Rolandic operculum	R	187	BA43	39	−3	15
Anterior cingulate cortex (ACC)	L	122	BA24	−6	35	1
Anterior cingulate cortex (ACC)	R	65	BA24	7	37	1

AN, anorexia nervosa; BA, Brodmann area; FC, functional connectivity; ACC, anterior cingulate cortex; L, left; R, right.

### Subgroup analyses

In the female-only group, the primary abnormal networks identified were the auditory network (22%), dorsal DMN (28%), and ventral DMN (12%) (Supplementary Figure S6). The adolescent group showed a broader distribution of abnormal networks, with predominant involvement of the auditory network (77%), LECN (79%), ventral DMN (92%), SMN (93%), precuneus network (90%), primary visual network (75%), and visuospatial network (49%) (Supplementary Figure S7). In the adult group, predominant abnormalities were found in the auditory network (92%), posterior salience network (60%), SMN (35%), and basal ganglia network (32%) (Supplementary Figure S8).

## Discussion

To the best of our knowledge, this study is the first to integrate the FCNM approach with large-scale resting-state fMRI data from the HCP, ultimately mapping GM changes in AN onto specific brain functional networks. Using the FCNM approach, we analyzed data from 26 VBM studies examining whole-brain GM alterations including a total of 667 individuals with AN and 659 healthy controls. The FCNM approach identified specific brain functional networks associated with GM alterations in AN, including the auditory network (e.g., bilateral superior temporal gyrus and right Rolandic operculum), the ventral and dorsal DMN (e.g., bilateral precuneus, bilateral ACC, and right middle temporal gyrus), and the sensorimotor network (SMN) (e.g., bilateral precentral gyrus). Network overlap was calculated using a 4-mm radius sphere, with validations using 1-mm and 7-mm sphere radii yielding robust and replicable results. Subgroup analyses showed that gender appeared to modulate the involvement of the SMN. Significant differences were also observed in the ECN, visual network, and salience network between adolescents and adults with AN. Notably, our findings offer a compelling bridge between neurobiology and the broader biopsychosocial framework of AN, suggesting the structural network vulnerabilities we identified serve as a crucial neural substrate upon which psychological traits and sociocultural pressures act to entrench the disorder.

### Auditory network alterations in AN

Our study found that GM alterations associated with AN spatially map onto the auditory network. The auditory network, as defined by the 14 canonical brain networks, encompasses the primary and secondary auditory cortices, superior temporal gyri, Rolandic operculum, and prefrontal cortex, is vital for processing environmental stimuli (53, 67). Auditory cortex dysfunction, characterized by altered FC, is implicated in body image distortion and interoceptive deficits in AN (68, 69). This dysfunction may reflect impaired integration of visual, auditory, and tactile stimuli, crucial for accurate body perception (70, 71). Reduced activity in the primary auditory cortex and insula is linked to abnormal bodily state perception (68). Furthermore, reduced left superior temporal gyrus volume in adolescents with AN correlates with weight and shape concerns (72, 73). Experiments manipulating footstep frequencies (high and low) further indicate that auditory signals, processed by the auditory cortex, may influence perceptions of body weight (69). Overall, the auditory cortex is crucial for multimodal sensory integration, and its functional abnormalities may distort the processing of auditory signals in AN patients, further exacerbating distortions in body image and weight perception (74, 75).

The superior temporal gyrus, including Heschl’s gyrus (the core of the primary auditory cortex), plays a key role in spectro-temporal analysis, phonological processing, and integrating cues for speech comprehension (76). It also contributes to processing social and emotional cues, which are often impaired in AN (77, 78). Studies show altered superior temporal gyrus activation in AN during exposure to food and body-image stimuli, with both hyper-activation and hypo-activation reported (79, 80). Decreased superior temporal gyrus activation when viewing food images may reflect diminished emotional engagement or reward processing (81, 82). The Rolandic operculum plays a key role in motor control related to oral and pharyngeal functions, taste perception, somatosensory processing, and multimodal integration (83). Given its involvement in taste perception and oral motor functions, the Rolandic operculum likely influences eating habits through its interactions with neural networks regulated by the hypothalamus (84). In individuals with AN, reduced GMV in the left Rolandic operculum has been linked to distortions in self-perception and social cognition (24).

### DMN alterations in AN

The DMN is traditionally categorized into the ventral DMN and the dorsal DMN, distinguished by anatomical and functional characteristics (53, 85). Key DMN regions include the precuneus, medial prefrontal cortex, posterior cingulate cortex, angular gyrus, hippocampus, and temporal and parietal areas (86). Our study found that the dysfunctional ventral DMN (middle temporal gyrus and precuneus) and dorsal DMN (ACC) were linked to GMV alterations in AN. The dorsal DMN plays a crucial role in assessing the emotional significance of envisioned scenarios, thereby impacting emotional processing and the development of prospective strategies (87), while the ventral DMN is mainly involved in the constructive process of imagination, assisting in combining memory fragments to create vivid and detailed mental images (85). DMN aberrations in AN, particularly affecting the precuneus, posterior cingulate cortex, and medial prefrontal cortex, are associated with distorted self-perception and intensified body image concerns (29, 88, 89). Additionally, increased DMN connectivity has been associated with heightened rumination on weight and body shape, suggesting that AN patients may experience intensified self-focused thoughts that perpetuate body image distortions (90, 91). Importantly, nutritional and psychological treatments have been shown to modulate abnormal DMN connectivity (30, 92), potentially restoring network function to support positive self-perception and body image, and reducing weight and body preoccupation in AN.

The middle temporal gyrus is essential for semantic cognition as it integrates information from the anterior temporal lobe and links it to goal-directed cognitive processes (93, 94). Studies have revealed structural and functional modifications in the middle temporal gyrus in patients with AN, which are closely related to symptoms including emotional dysregulation and body weight control (95–97). For instance, AN patients often experience a decrease in GMV in the middle temporal gyrus, which is associated with symptoms of body dissatisfaction and distorted self-image (19, 98). Functional MRI studies indicate modified activation in the middle temporal gyrus during tasks related to body, food, and cognitive processing, which could potentially worsen symptoms of AN (98, 99). Furthermore, reduced activation in the middle temporal gyrus is linked to impaired emotional recognition and theory of mind, impacting social interactions and self-perception in those with AN (100, 101).

The precuneus, part of the posterior DMN, is associated with self-referential processes and body awareness, functions that are often disrupted in AN (98). Structural changes like cortical thinning and GMV have been observed, sometimes reversing with weight restoration (102, 103). Altered precuneus resting-state FC, particularly with the ACC, correlates with body image concerns, emotional regulation difficulties, and distorted body perception (104–106). The ACC is essential for integrating emotion, motivation, and cognitive control to support goal-directed behavior, decision-making, and adaptive responses to rewards and punishments (107). The ACC plays a significant role in the pathophysiology of AN, with studies demonstrating both structural and functional alterations that relate to core symptoms of the disorder (10, 77). For instance, research has shown consistent gray matter reduction in the ACC of AN patients, even after weight recovery, suggesting long-lasting structural deficits associated with the severity of the disorder (63, 108). Resting-state studies further reveal disrupted synchrony between the ACC and regions like the precuneus, which correlates with concerns about body shape, highlighting a network-level dysfunction underlying the obsessive focus on body image (106).

### SMN alterations in AN

The SMN, as defined by the 14 canonical brain networks, encompasses the precentral gyrus, postcentral gyrus, and supplementary motor area, plays a crucial role in integrating and processing sensory and motor information within the brain (109–111). We determined that the altered SMN associated with GMV changes in AN involved the precentral gyrus. Research indicates that AN patients exhibit abnormal connectivity within the SMN, especially in areas related to the integration of somatosensory and visuospatial information (29, 102). The dysfunction of the SMN in AN is linked to impaired body image perception and sensory processing, which are central to the disorder’s pathology (68, 112). The biological underpinnings of body image distortions in AN patients involve impaired sensorimotor integration and disrupted FC (21, 113).

The precentral gyrus is essential for voluntary body movement control, motor planning, and coordination (114, 115). Neuroimaging has shown altered FC involving the precentral gyrus in AN patients, often associated with behaviors regarding food intake and body image (21, 116). Moreover, studies have found decreased GMV in the precentral gyrus of AN patients, a structural alteration that might underpin difficulties in flexible motor responses and contribute to the disorder persistent behavioral patterns (11, 117). These findings highlight the precentral gyrus role in the neurobiological mechanisms underlying AN and suggest that both functional and structural disruptions in this area may reinforce the motor control and body perception challenges characteristic of the disorder (88, 118).

### Subgroup analyses in AN

When considering the female-only subgroups (24 studies), patients with AN primarily exhibited significant alterations in the auditory network, dorsal DMN, and ventral DMN, with comparatively lesser involvement observed for the SMN. Research has demonstrated anatomical and functional differences in the sensorimotor cortex between males and females, especially in the precentral and postcentral gyri (110). In females, these regions are generally more involved in emotional processing and bodily self-awareness, whereas males show a stronger association with motor control functions (119). A typical symptom of AN is an intense preoccupation with weight and body image (120). Women are typically more vulnerable than men to the sociocultural pressures that promote the thin ideal, which leads to a more pronounced body image distortion (121). Given the close connection between the SMN and bodily self-awareness, female patients may experience more significant impairments in network functionality because of enhanced body image distortion (122). Additionally, among AN patients, emotional disturbances such as anxiety and depression are more prevalent in females and are closely linked to dysfunctions in the sensorimotor network (123).

Our study reveals that the ECN, visual network, and salience network are influenced by age in patients with AN. Research indicates that the development of the prefrontal cortex, essential for executive functions, continues during adolescence, possibly resulting in weaker executive control, emotional regulation, and social skills in adolescents compared to adults (27, 124). In adolescents, the still-developing prefrontal cortex may relate to increased impulsivity and emotional instability when faced with emotional and social challenges that require self-regulation (125). Furthermore, the salience network and visual network exhibit different patterns of activity in adolescent and adult AN patient. The salience network in adolescents shows greater plasticity and heightened responsiveness to environmental stimuli (126). In contrast, the functioning of these networks in adult AN patients may be more stable (127).

### Limitations

There are several limitations to this study. First, the analysis relies on coordinate-based data extracted from published studies, which offers a summary statistic (peak location) and inherently lacks spatial information in comparison to analyses using full statistical maps or individual participant data (40, 42, 128). This coordinate-based approach is correlational and does not have the capacity to determine causality between the identified network and AN-related GM alterations. Second, although the FCNM approach has effectively mapped neuropsychiatric symptoms and diseases onto common brain networks, we utilized resting-state fMRI data from a large cohort of healthy adults provided by the HCP. It may be more suitable to employ data that aligns more closely with the demographic and clinical profiles of the patients included in the analyzed studies. Third, due to the limited number of previous studies, network localization analyses were not conducted separately for different stages of AN (acute, chronic, and recovered). Future studies should address this by performing stage-specific network localization analyses to enhance result precision. Fourth, since many individuals with AN are also treated with antidepressants, it creates a challenge in distinguishing whether the brain network abnormalities observed are a result of AN itself or are influenced by the medications. This is a common issue in clinical research, as medications may alter brain function, potentially confounding the results. It highlights the need for more controlled studies that separate the effects of AN from those of psychotropic treatments. Fourth, while the FCNM approach is effective for mapping GM abnormalities to specific brain networks, it is constrained by the spatial resolution and methodological limitations of neuroimaging techniques. This limitation may lead to an underestimation of network-level abnormalities as subtle changes in smaller or overlapping network regions may not be fully captured. Additionally, the current study combined coordinates representing both increases and decreases in GM, which might obscure network specificities related to the direction of change. Future research should focus on validating these findings in clinical cohorts, exploring the longitudinal trajectories of brain network alterations, and assessing their potential as biomarkers for treatment response and prognosis. By combining advanced imaging techniques with network analysis methods, a more comprehensive understanding of the complex neurobiological mechanisms underlying AN can be achieved, providing new scientific evidence for targeted therapeutic interventions.

## Conclusion

In conclusion, this study integrated the FCNM approach and large-scale human brain connectome data obtained from the HCP, revealing that the heterogeneous GM abnormalities in AN converge onto specific brain networks. Our findings indicated that the aberrant brain networks linked to AN predominantly implicate the auditory network (bilateral superior temporal gyrus and right Rolandic operculum), the ventral DMN (right middle temporal gyrus and bilateral precuneus), the dorsal DMN (bilateral ACC), and the SMN (bilateral precentral gyrus). Furthermore, network abnormalities in AN are influenced to some extent by both gender and age. These disruptions in brain networks are associated with distorted body image perception, impaired emotional regulation, and disrupted sensory and motor integration in individuals with AN. Network localization offers a comprehensive and unified framework that may help address concerns regarding the reproducibility of GM findings across VBM studies in AN. These findings could enhance our comprehension of the pathological mechanisms that underlie AN from a network perspective.

## Data Availability

The original contributions presented in the study are included in the article/Supplementary material, further inquiries can be directed to the corresponding authors.

## Footnotes

^1^ https://www.sdmproject.com

^2^ https://www.fil.ion.ucl.ac.uk/spm/

^3^ https://rfmri.org/DPABI

## Glossary

ACC: anterior cingulate cortex
AN: anorexia nervosa
CBMA: coordinate-based meta-analysis
DBM: deformation-based morphometry
DMN: default mode network
FA: flip angle
FC: functional connectivity
FCNM: functional connectivity network mapping
FDR: false discovery rate
fMRI: functional magnetic resonance imaging
FOV: field of view
FWE: family-wise error
FSL: FMRIB Software Library
FWHM: Full Width at Half Maximum
GRE-EPI: gradient-recalled echo-Planar Imaging
GM: gray matter
GMV: gray matter volume
HC: healthy controls
HCP: Human Connectome Project
LECN: left executive control network
MRI: magnetic resonance imaging
MNI: Montreal Neurological Institute
NA: not applicable
ROI: region of interest
RECN: right executive control network
SMN: sensorimotor network
SPM: Statistical Parametric Mapping
TE: echo time
TFCE: Threshold-Free Cluster Enhancement
TR: repetition time
VBM: Voxel-based morphometry.
