# Supplementary material for: Mapping gray matter changes in anorexia nervosa: a functional connectivity network approach
Source: Front Nutr. 2025 Sep 17;12:1667729. doi: 10.3389/fnut.2025.1667729 (PMC12486608; doi:10.3389/fnut.2025.1667729)
Supplement: Supplementary file 1 [file Data_sheet_1.docx]

**Supplementary Information**

**Mapping grey matter changes in anorexia nervosa: A functional connectivity network approach**

**Table S1.** **Quality evaluation checklists**

**Category 1: Participants**

1. Patients were evaluated prospectively, certain diagnostic criteria were used, and demographic characteristics were reported.

2. Healthy controls were evaluated prospectively, psychiatric and medical diseases were excluded.

3. Essential variables (e.g., age, gender, illness duration, symptom severity, family history, medication) were checked either by stratification or statistics.

4. Both male and female participants were included and sample size in each group > 10.

**Category 2: Methodology for image acquisition and process**

5. Whole-brain level analysis was automated with no priori selection of regions.

6. Spatial coordinates were reported in a standard space (e.g., Talairach or MNI coordinates)

7. The imaging techniques utilized were clearly described for reproducibility.

8. Measurements were clearly described for reproducibility.

**Category 3: Results and conclusions**

9. Statistical parameters for both significant and critical non-significant differences were reported.

10. Conclusions were consistent with the results and the limitations were discussed.

Score 0/0.5/1 per item; Total score out of 10; For criteria partially met, 0.5 points were given.

MNI, Montreal Neurological Institute

**Table S2. Demographic information of the HCP**

| **Dataset sample size** | **Age (years)** | **Gender (F/M)** |
| --- | --- | --- |
|  |  |  |
| HCP 1093 | 28.78±3.69 | 594/499 |

Age is expressed as mean ± standard deviation. Note: HCP, Human Connectome Project; F, female;

M, male.

**Table S3. Resting-state fMRI parameters of the HCP**

| **Parameter** | **HCP** |
| --- | --- |
| Scanner | 3.0T Siemens Trio |
| Sequence | GRE-EPI |
| TR (ms) | 720 |
| TE (ms) | 33.1 |
| FA (°) | 52 |
| FOV (mm²) | 208×180 |
| Matrix size | 104×90 |
| Slice thickness (mm) | 2 |
| Slice gap (mm) | 0 |
| Slices | 72 |
| Time points | 1210 |

HCP, Human Connectome Project; GRE-EPI, gradient-recalled echo-Planar Imaging; FA, flip angle; fMRI, functional magnetic resonance imaging; FOV, field of view; TE, echo time; TR, repetition time.


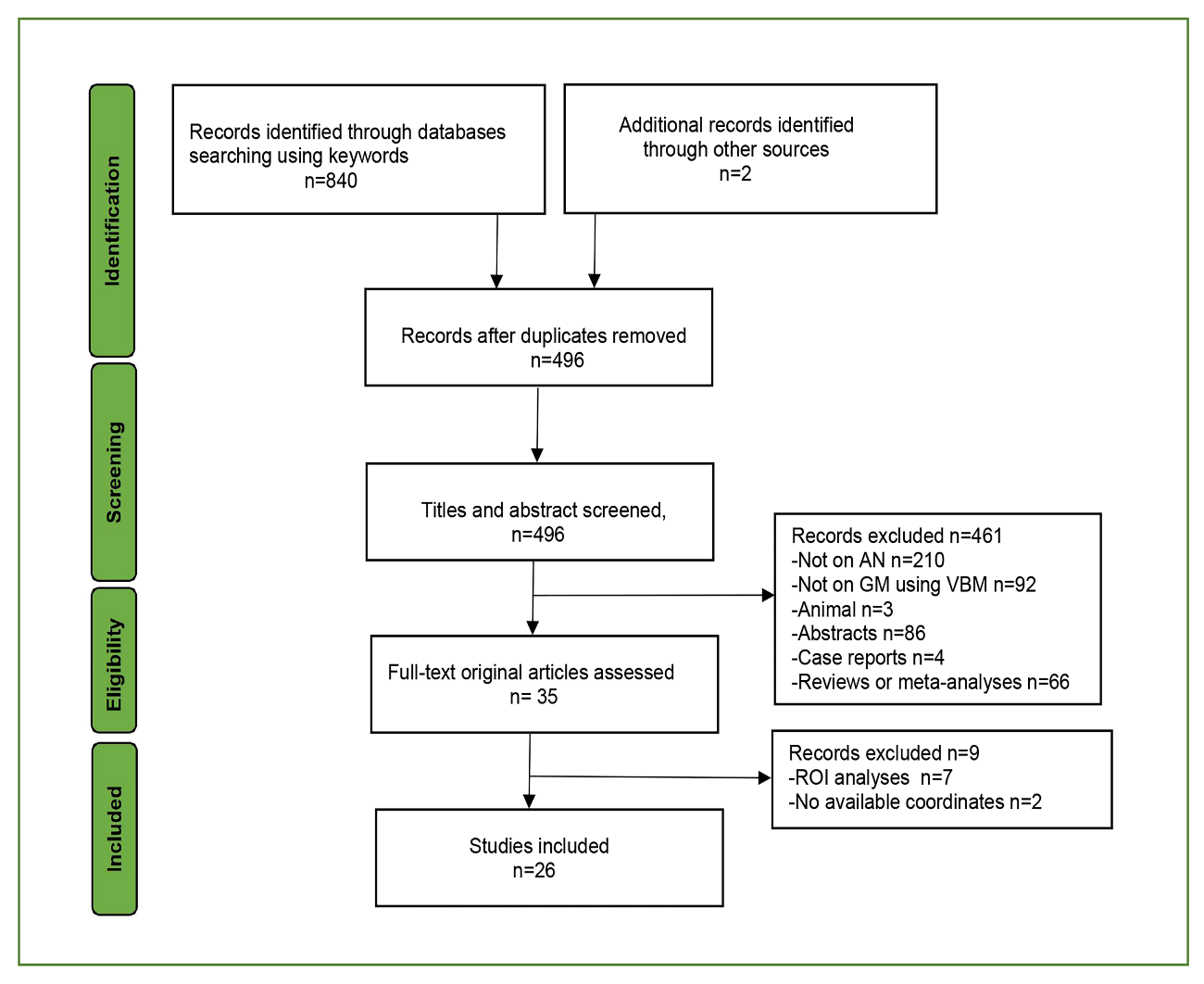
**Figure S1.** **A flow diagram of the study selection process.**

AN, anorexia nervosa; GM, gray matter; VBM, voxel-based morphometry; ROI, region of interest.


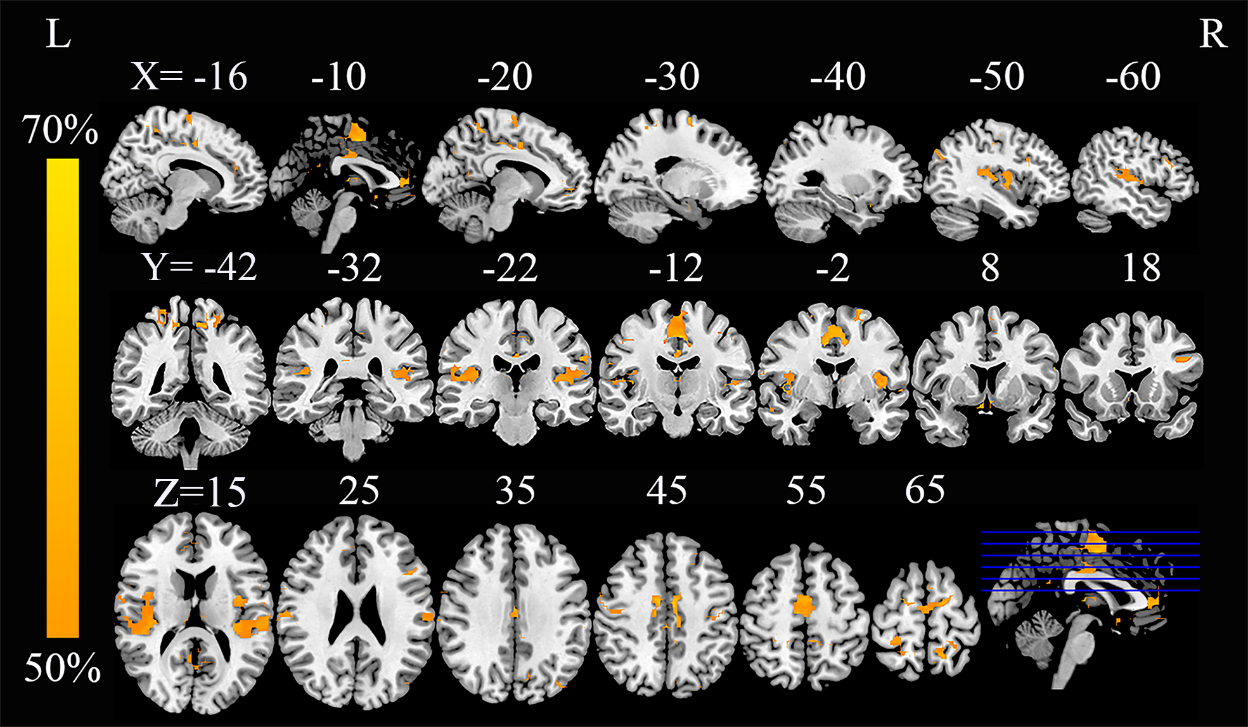


**Figure S2.** **FC overlap maps based on 1-mm radius sphere in AN.** Dysfunctional brain networks are shown as FC probability maps thresholded at 50%, showing brain regions functionally connected to more than 50% of the contrast seeds.AN, anorexia nervosa; FC, functional connectivity; L, left; R, right.


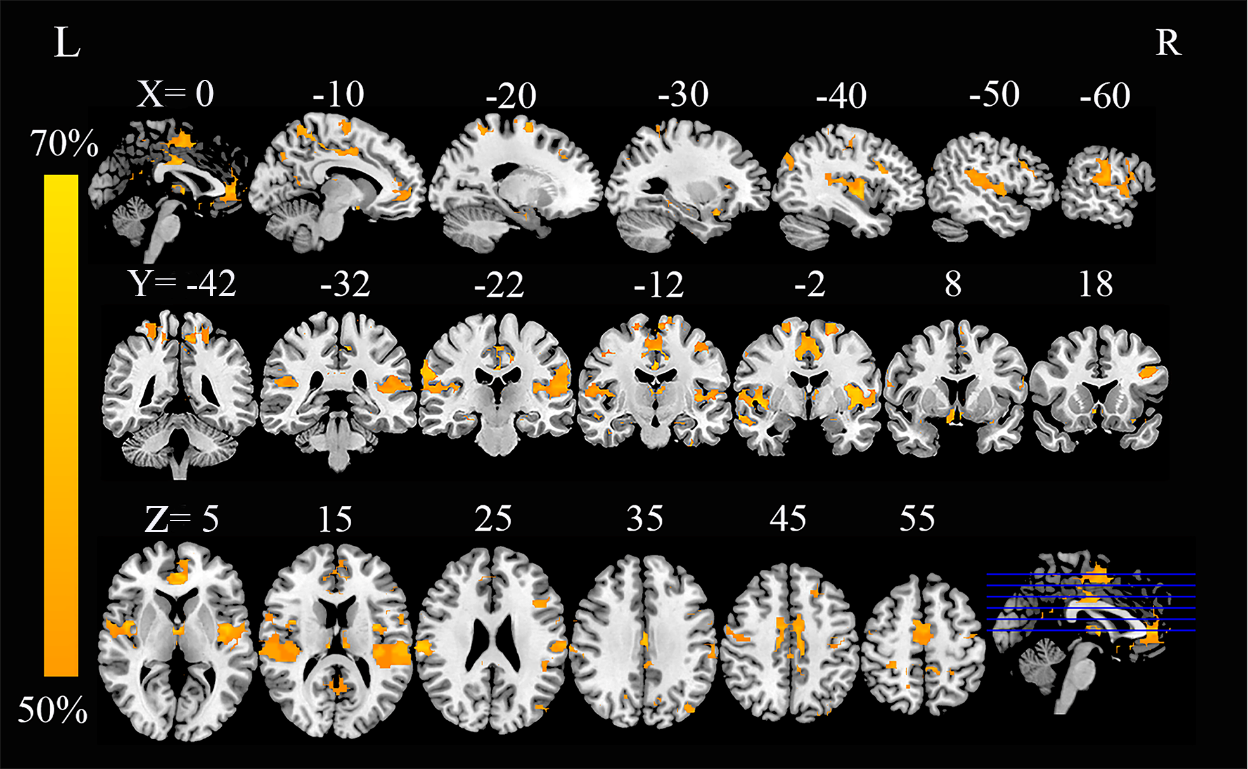


**Figure S3. FC overlap maps based on 7-mm radius sphere in AN.** Dysfunctional brain networks are shown as **FC** probability maps thresholded at 50%, showing brain regions functionally connected to more than 50% of the contrast seeds.AN, anorexia nervosa; FC, functional connectivity; L, left; R, right.


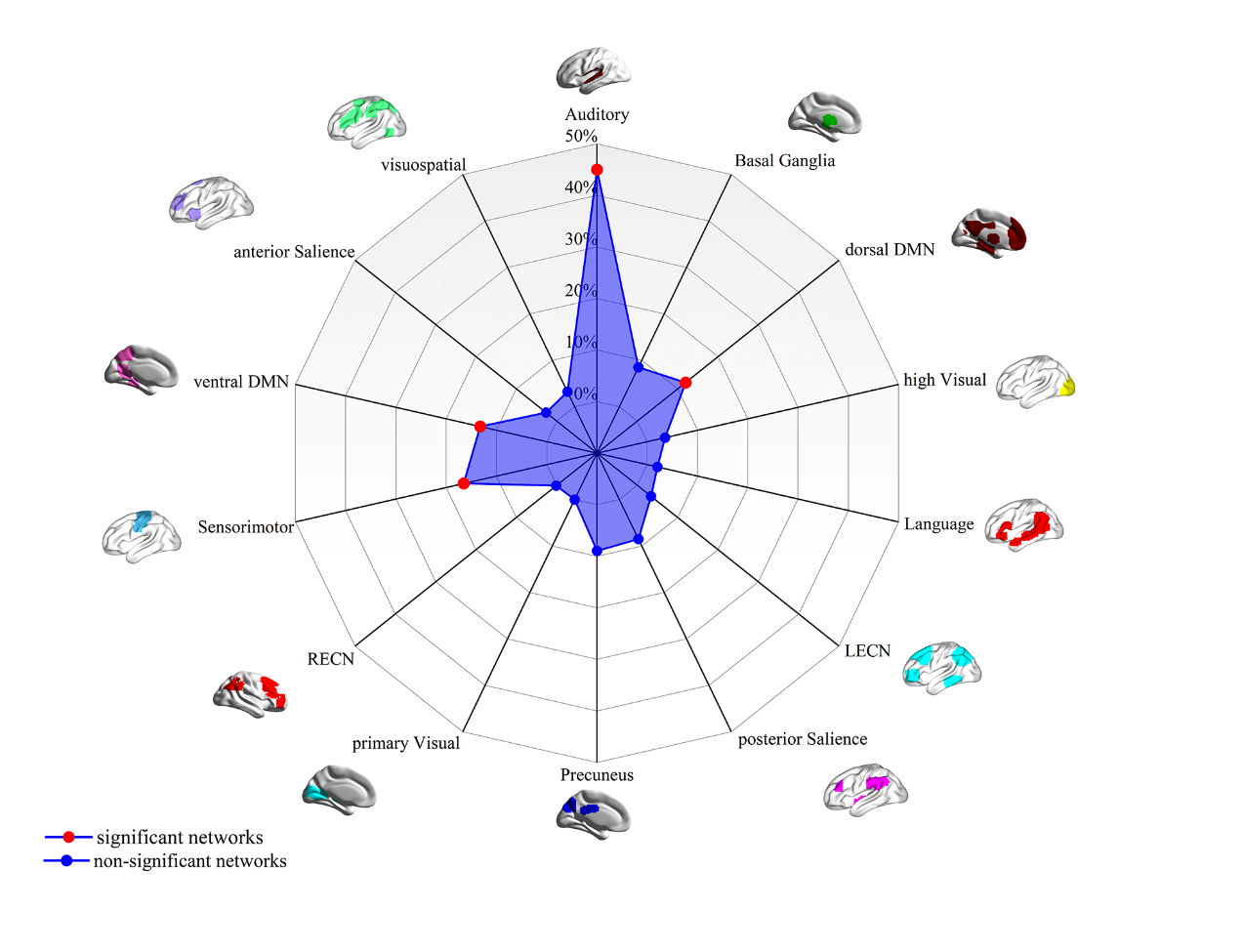


**Figure S4. FC overlap maps based on 1-mm radius sphere in association with canonical brain networks.** Polar plots illustrate the proportion of overlapping voxels between each AN dysfunctional network and a canonical network to all voxels within the corresponding canonical network. Note: The red dot represents brain dysfunction networks, defined as significant networks, exhibiting ≥ 10% overlap with canonical networks, whereas the blue dot represents non-significant networks with <10% overlap.

DMN, default mode network; FC, functional connectivity; LECN, left executive control network; RECN, right executive control network.


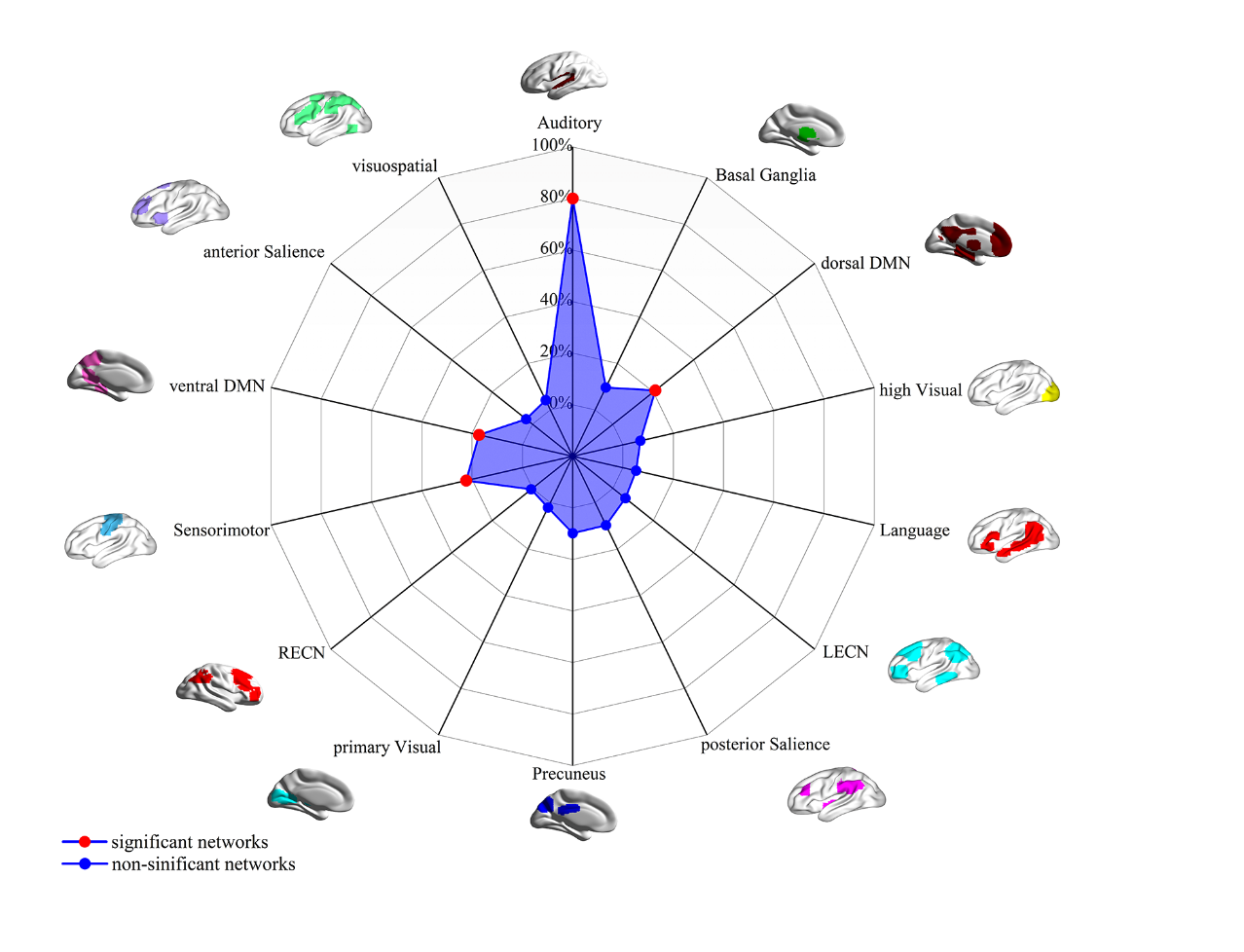


**Figure S5. FC overlap maps based on 7-mm radius sphere in association with canonical brain networks.** Polar plots illustrate the proportion of overlapping voxels between each AN dysfunctional network and a canonical network to all voxels within the corresponding canonical network. Note: The red dot represents brain dysfunction networks, defined as significant networks, exhibiting ≥ 10% overlap with canonical networks, whereas the blue dot represents non-significant networks with <10% overlap.

DMN, default mode network; FC, functional connectivity; LECN, left executive control network; RECN, right executive control network.


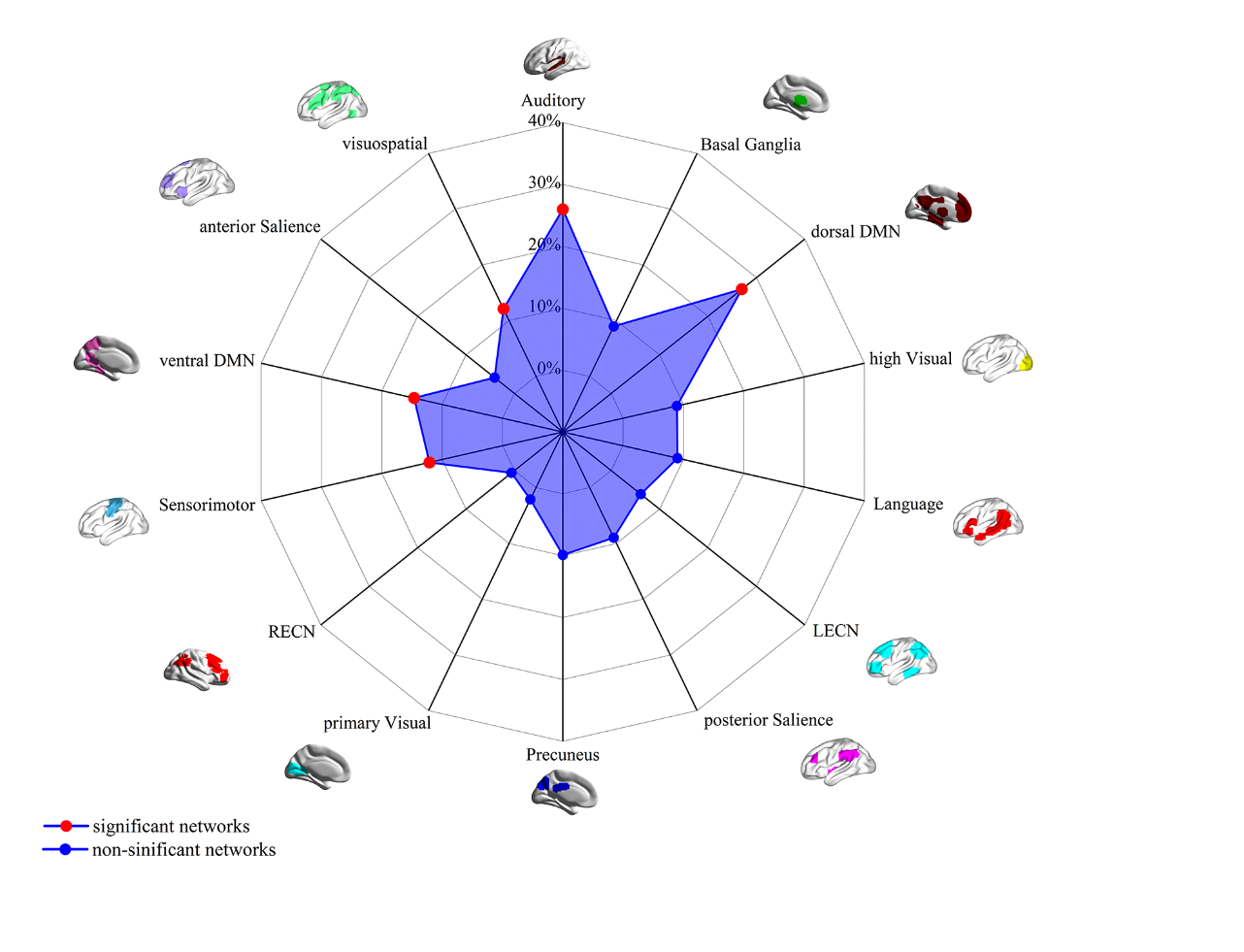


**Figure S6.** **FC overlap maps based on 4-mm radius sphere in association with canonical brain networks (female-only subgroup).** Polar plots illustrate the proportion of overlapping voxels between each AN dysfunctional network and a canonical network to all voxels within the corresponding canonical network. Note: The red dot represents brain dysfunction networks, defined as significant networks, exhibiting ≥ 10% overlap with canonical networks, whereas the blue dot represents non-significant networks with <10% overlap.

DMN, default mode network; FC, functional connectivity; LECN, left executive control network; RECN, right executive control network.

**
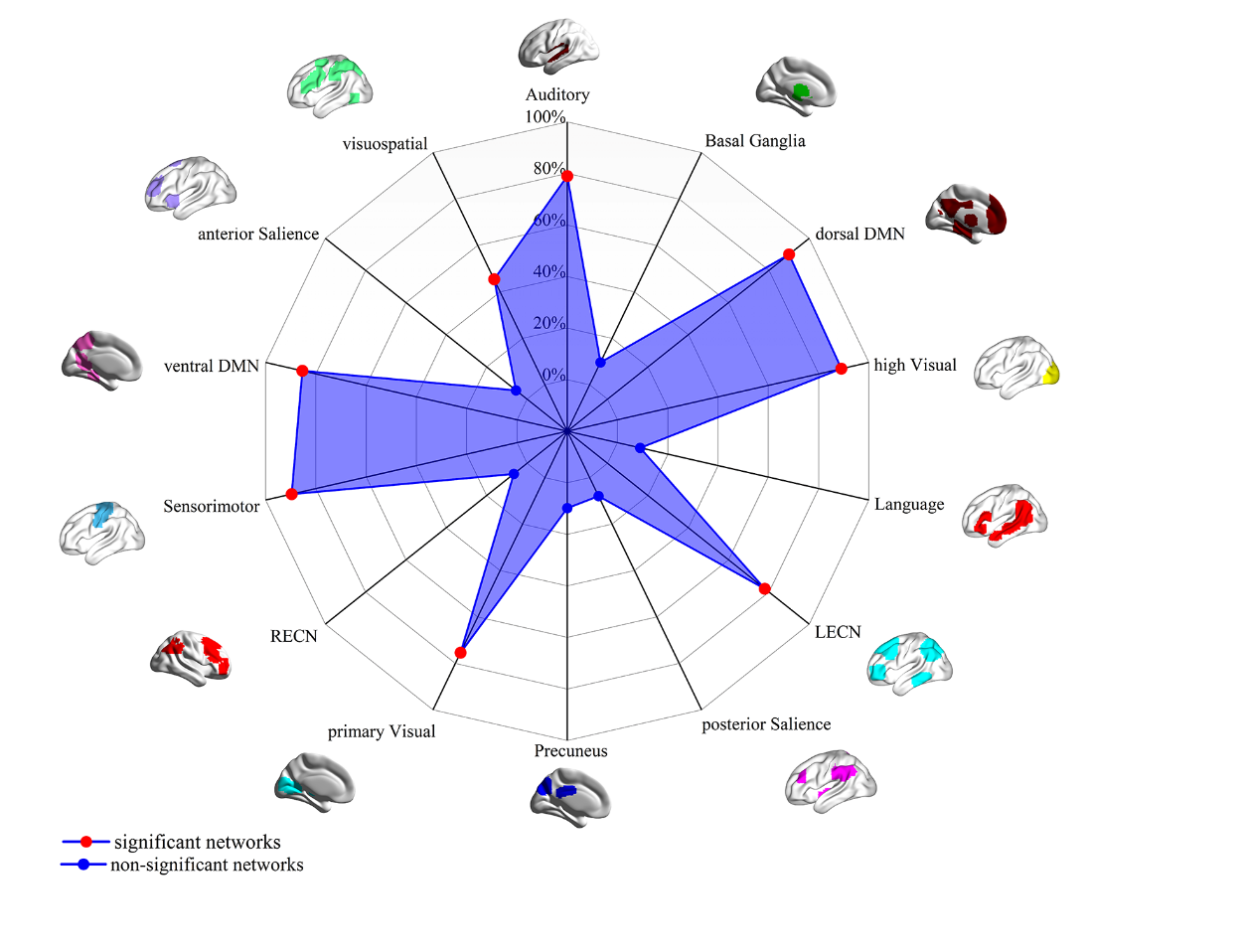
**

**Figure S7.** **FC overlap maps based on 4-mm radius sphere in association with canonical brain networks** **(adolescent subgroup).** Polar plots illustrate the proportion of overlapping voxels between each AN dysfunctional network and a canonical network to all voxels within the corresponding canonical network. Note: The red dot represents brain dysfunction networks, defined as significant networks, exhibiting ≥ 10% overlap with canonical networks, whereas the blue dot represents non-significant networks with <10% overlap.

DMN, default mode network; FC, functional connectivity; LECN, left executive control network; RECN, right executive control network.


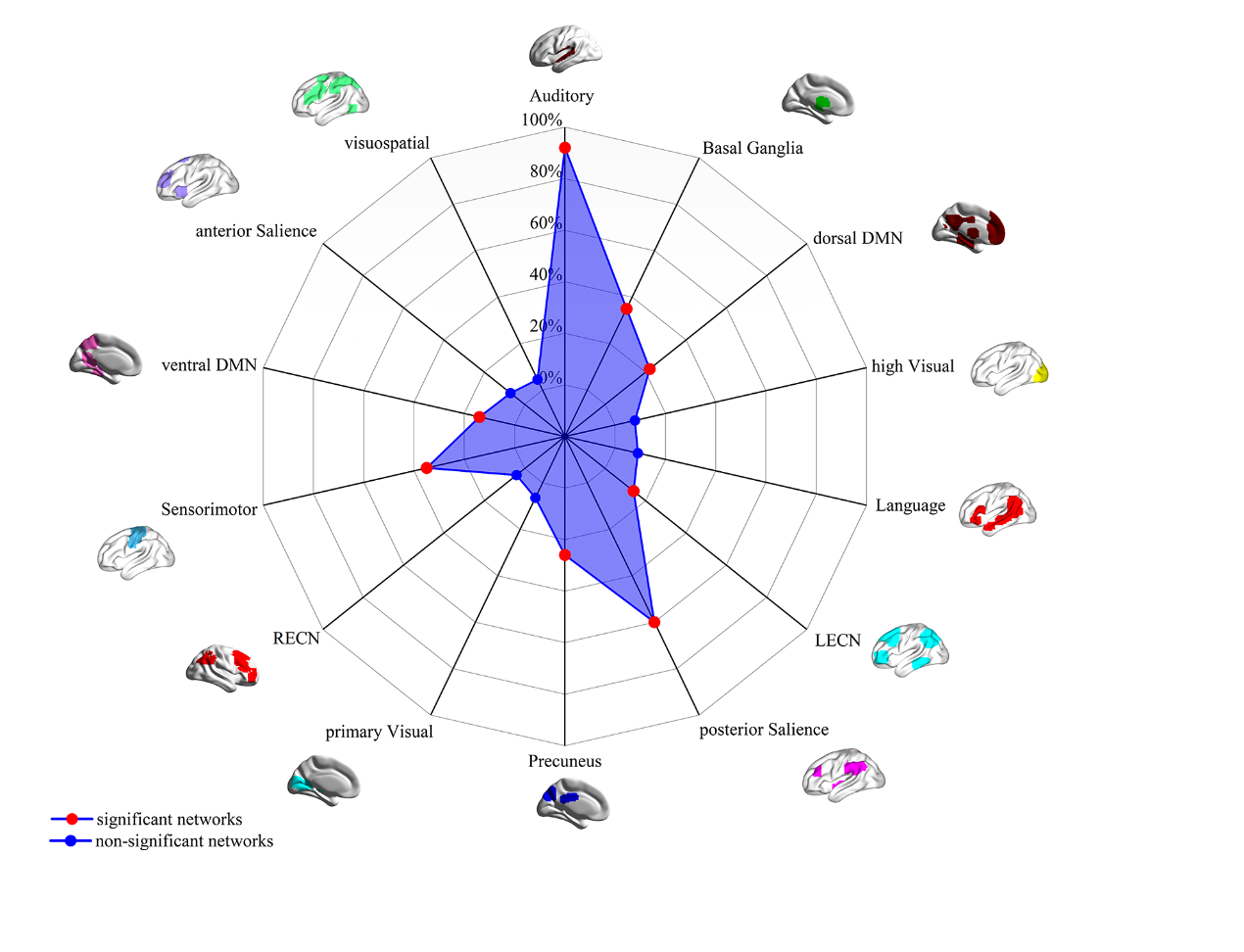


**Figure S8. FC overlap maps based on 4-mm radius sphere in association with canonical brain networks** **(adult subgroup).** Polar plots illustrate the proportion of overlapping voxels between each AN dysfunctional network and a canonical network to all voxels within the corresponding canonical network. Note: The red dot represents brain dysfunction networks, defined as significant networks, exhibiting ≥ 10% overlap with canonical networks, whereas the blue dot represents non-significant networks with <10% overlap.

DMN, default mode network; FC, functional connectivity; LECN, left executive control network; RECN, right executive control network
